# Regulation of the Target Protein (Transgene) Expression in the Adenovirus Vector Using Agonists of Toll-Like Receptors

**Published:** 2014

**Authors:** A. V. Bagaev, A. V. Pichugin, E. S. Lebedeva, A. A. Lysenko, M. M. Shmarov, D. Yu. Logunov, B. S. Naroditsky, R. I. Ataullakhanov, R. M. Khaitov, A. L. Gintsburg

**Affiliations:** National Research Center – Institute of Immunology Federal Medical-Biological Agency of Russia; Kashirskoye shosse, 24, corpus 2, 115478, Moscow, Russia; N.F. Gamaleya Research Institute of Epidemiology and Microbiology, Ministry of Health of the Russian Federation; Gamaleya Str., 18, 123098, Moscow, Russia

**Keywords:** gene therapy, gene vaccination, recombinant replication-defective adenovirus vectors, transgene expression, Toll-like receptor agonists

## Abstract

Replication-defective adenoviral vectors are effective molecular tools for both gene therapy and gene vaccination. Using such vectors one can deliver and express target genes in different epithelial, liver, hematopoietic and immune system cells of animal and human origin. The success of gene therapy and gene vaccination depends on the production intensity of the target protein encoded by the transgene. In this work, we studied influence of Toll-like receptors (TLR) agonists on transduction and expression efficacy of adenoviral vectors in animal and human antigen-presenting cells. We found that agonists of TLR2, 4, 5, 7, 8 and 9 significantly enhance a production of the target protein in cells transduced with adenoviral vector having the target gene insert. The enhancement was observed in dendritic cells and macrophages expressing cytoplasmic (GFP), membrane (HA) or secretory (SEAP) proteins encoded by the respective rAd-vectors. Experiments in mice showed that enhancement of the transgene expression can be achieved in the organism of animals using a pharmaceutical-grade TLR4-agonist. In contrast to other TLR-agonists, the agonist of TLR3 substantially suppressed the expression of transgene in cells transduced with adenoviral vectors having insert of GFP or SEAP target genes. We propose that the enhancement of transgene expression is linked to the activation of MyD88→ NF-kB, while the inhibition of transgene expression depends on TRIF→ IRF signaling pathways. Both of these pathways jointly exploited by TLR4-agonists lead to the enhancement of transgene expression due to the dominant role of the MyD88→ NF-kB signaling.

## INTRODUCTION

Replication-defective adenovirus vectors (rAd) are used for transgene expression in different tissues. Depending on the transgene inserted rAd are used for gene therapy (tumor suppressor genes, growth factors, etc) or gene immunization (genes encoding infection- specific antigens). In the case of in vivo immunization, the transgene is expressed in transduced cells during 2-3 weeks, which leads to a potent immune response. Effective vaccines against tuberculosis, malaria, influenza, and other important infectious diseases have been designed as rAd. The majority of rAd-based medical preparations are currently in various stages of clinical trials [1-5], and only one therapeutic agent has been licensed and approved for use [6]. There is a large body of evidence demonstrating that rAd-derived immunogens and viccines are safe and efficacious.

The immune response to the rAd-expressed antigen could be enhanced by Toll-like receptor agonists (TLR) [3]. We engineered a rAd-based vaccine encoding the surface antigens of the influenza virus in combination with a synthetic TLR4 agonist – Immunomax® [7-9], a plant-derived water soluble polysaccharide of a molecular weight of over 1 MDa [10]. The use of Immunomax as an adjuvant enhances the T-cell response to Influenza virus antigens and increases rAd-vaccine protectivity. In addition, this adjuvant allows a 3- to 10- fold reduction in the dosage of rAd, encoding influenza virus antigens, without compromising immunogenicity and protective efficacy of the vaccine.

Immunomax has no direct influence on T-cells. It stimulates antigen-presenting cells, particularly dendritic cells and macrophages. In dendritic cells, Immunomax induces the expression of co-stimulatory molecules such as CD80, CD86, CD40, and the MHC class II and the release of immunostimulatory cytokines such as IL1β, TNF-α, IL6, IL8, and IL12 [10]. Although these events in antigen-presenting cells are sufficient for the immunostimulatory response elicited by Immunomax, it is likely that Immunomax also increases the expression of antigen encoded by rAd.

Following administration of rAd, the antigen is produced in antigen-presenting cells, which allows one to suppose that Immunomax, alongside co-stimulatory molecules and cytokines, could also promote the expression of rAd-encoded antigen. Such stimulatory activity of the preparation could contribute to an enhanced response of T-cells, recognizing antigens on the surface of the antigen-presenting dendritic cells.

In the present study, we examined the expression levels of the rAd-encoded antigens in dendritic cells and macrophages treated with Immunomax and agonists of other TLRs. The findings demonstrate that under influence of Immunomax the expression of rAdencoded antigens in dendritic cells and macrophages is elevated 2- to 11-times. The increased expression was observed with rAd-encoded membrane-bound, cytoplasmic, and secretory proteins. Similarly to Immunomax, another TLR4-agonist --- an *Escherichia coli *Lipopolysaccharide --- also up-regulated the expression of rAd-encoded transgenic proteins. In addition, agonists of other TLRs, in particular TLR2, 5, 7, 8 and 9, increased the expression levels of rAd-encoded proteins, whereas the TLR3 agonist down-regulated it. Comparison of the intracellular signaling pathways linked to different TLRs suggested that the pathway beginning from MyD88 and ending with NF-κB does enhance expression, but the pathway beginning from TRIF and ending with the IRF-3 and IRF-7 transcription factors inhibits the expression of rAd-encoded proteins. The pathway started by MyD88 dominates over the one started by TRIF. Therefore, transgene expression is increased under influence of TLR4 agonist, when both pathways are operational (MyD88 and TRIF).

## EXPERIMENTAL SECTION

**Antibodies and reagents**

The following monoclonal antibodies were used: CD11b-BD Horizon V450, Ly-6G APC-Cy7 (BD Pharmingen™), CD11c PE, CD19 eFluo450 (eBiosciences), F4/80 APC (BioLegend), Anti Mouse MHC II (I-A)- FITC (eBiosciences). Monoclonal antibodies to H1N1 influenza virus (HA) were a courtesy of A.A. Kushch (Ivanovskii Institute of Virology, Ministry of Health of the Russian Federation). The following Toll-like receptor agonists were used: lipoteichoic acid (LTA, TLR2 ligand), a synthetic analog of double-stranded RNA – polyinosinic:polycytidylic acid (Poly[I:C], TLR3 ligand), monophosphoryl lipid A (MPL-A, TLR4 ligand), flagellin (TLR5 ligand), imiquimod (TLR7/8 ligand), synthetic oligonucleotide ODN-CpG 1826 (TLR9 ligand), all obtained from InvivoGen; lipopolysaccharide from *E. coli *serotype 055:B5 (LPS, TLR4 ligand, Sigma, L-2880). In addition, a recombinant tumor necrosis factor alpha was used (TN F-α, Sigma, T7539). In some experiments CLI-095 (InvivoGen), a specific inhibitor of the TLR4-dependent pathway, DAPI dihydrochloride (Sigma), was used as a nuclear counterstain**.**

**Animals**

Eight- to ten-week-old BALB/c mice were obtained from the breeder Stolbovaya and fed standard rodent food under standard animal house conditions in the vivarium of the National Research Center Institute of Immunology FMBA.

**Cell cultures**

All cell cultures were incubated in a complete medium (CM) based on DMEM with 25 mM HEPES supplemented with a cocktail of nonessential amino acids, 10% fetal bovine serum (FBS), 2 mM L- glutamine, 1 mM sodium pyruvate, 50 μM β-mercaptoethanol and 10 μg/ml gentamycin (all reagents obtained from PanEco) at 37°C in a 5% CO_2_ humidified atmosphere.

The cell line 293/TLR4-MD2-CD14 (InvivoGen), stably transfected with the TLR4 and CD14 and MD-2 co-receptors, was maintained *in vitro *in the presence of the selective antibiotics blasticidin and hygromycin according to the manufacture’s instructions. For the assessment of NF-κB activity, the cells were transduced with a lentivirus vector (Cleveland BioLabs) bearing a reporter β- galactosidase gene under the control of the NF-κB-dependent promoter. Thereafter, the cells were cultured following the manufacturer’s protocol in the presence of another selective antibiotic – puromycin.

Primary cell suspensions from the spleen, bone marrow, and peritoneal cavity were made as described elsewhere. Dendritic cells were obtained *in vitro *by culturing bone marrow cells of BALB/c mice with a granulocyte-macrophage colony-stimulating factor (GM-CSF). Bone marrow was washed out from the femurs and the tibias, erythrocytes removed by osmotic shock, nuclear cells washed twice in PBS ( Amresco, E404), followed by cultivation in a complete medium supplemented with 10 ng/ml GM-CSF (Sigma) for 7 days as described [4]. After 7 days of culture, the nonadherent cells contained 70–75% of dendritic cells. The adherent cells comprised 95% of macrophages. After removed of non-adherent cells and washing the remaining confluent cells with PBS (0.5% FBS), macrophages were detached by incubation in a Versen solution (PanEco) for 1 h at 4°C. Then cells were gently washed off in PBS (0.5% FBS).

Peritoneal macrophages were obtained by washing of the peritoneal cavity of BALB/c mice with PBS supplemented with 1% glucose, 10 mM HEPES, and 0.5% FBS. The cells were pelleted by centrifugation, resuspended in CM, and cultured for 18–20 h at 37°C in a humidified atmosphere of 5% CO_2_. Then non-adhesive cells were gently washed away with the medium and PBS. The remaining adherent cells comprised over 90% of macrophages.

**Recombinant replication-defective adenovirus vector with a gene insert**

Replication-defective adenovirus vectors rAd-SEAP, rAd-GFP, and rAd-HA carrying the gene of secreted embryonic alkaline phosphatase (SEAP), green fluorescent protein (GFP), or H1N1 influenza virus hemagglutinin (HA) were constructed based on the pShuttle- CMV plasmid according to the manufacturer’s instruction for the AdEasy Adenoviral vector system (Stratagene, cat. 240009), using the plasmids pGREEN (Carolina Biological Supply Company), p310D (pRc- CMV-SEAP, produced in-house) and pAL-HA (produced in-house). The GFP, SEAP, and HA inserts in the corresponding constructs pShuttle-CMV-GFP, pShuttle-CMV-SEAP, and pShuttle-CMV-HA were verified by restriction analysis using EcoRI, NotI and EcoRV endonucleases, and PCR.

The presence of the genes GFP, SEAP, and HA in rAd was confirmed by PCR. The effective titer of the rAd-GFP, rAd-SEAP, and rAd-HA preparations was estimated using the plaque-forming assay in the HEK- 293 cell culture [11].

**Transduction of cells with recombinant replication-defective adenovirus vectors**

The cell cultures were transduced with rAd-SEAP, rAd-GFP, or rAd-HA at a dose of 7–200 PFU/cell in a 50 μl OpTmizer™ serum-free medium (“GIBCO”) for 1 h, 150 μl of CM was then added to the cells. The transduced cells were cultured in the presence or absence of the TLR4 agonist Immunomax (10 μg/ml) for 1–6 days. When other TLR agonists were studied, the cells were incubated in the presence of LTA (1 μg/ml), Poly[I:C] (10 μg/ml), LPS (10 μg/ml), MPL-A (5 μg/ml), flagellin (0.1 μg/ml), imiquimod (1 μg/ml), or ODN-CpG 1826 (10 ng/ml). In addition, for cell activation, bypassing the TLR pathway, TNF-α (10 ng/ml) was used. TLR4- associated signaling was inhibited using CLI-095 (1 μg/ml).

**In vivo transduction with recombinant replication-incompetent adenovirus vectors**

For *in vivo *transduction, BALB/c mice were intraperitoneally injected with rAd-SEAP at a dose of 108 PFU in 200 μl of a physiological saline without or together with 10 μg Immunomax (four mice in each group).

Transgene expression rate was evaluated by the serum level of SEAP protein. On day 3 post-injection, blood was sampled from the retro-orbital sinus of rAd- SEAP-injected mice and serum level of SEAP was measured.

**Measurement of production intensity of the SEAP, GFP, and HA proteins encoded by rAd.**

The expression rates of SEAP in the serum or culture medium was assessed as described in [12] with minor modifications. Samples were clarified by centrifugation at 14,000 g for 2 min, followed by heating at 65oC for 5 min. The substrate p-nitrophenyl-phosphate was added in the reaction buffer (0.5 M CaCO3, 0.5 mM MgCl2, pH 9.8); and the absorbance was measured at 405 nm. The SEAP activity was expressed as mU/ml, given that 1 mU/ml corresponds to an increase in absorbance of 0.04 U/min.

Intracellular GFP accumulation was estimated by flow cytometry on FACS Aria II (BD Biosciences). Fluorescence was excited with the 488 nm laser, and emission intensity was measured between 515 and 545 nm. Cell populations were identified using fluorochromeconjugated antibodies to the surface proteins CD11b , CD11c, CD19, Ly6G, F4/80; followed by analysis on a FACS Aria II flow cytometer. In addition, accumulation of GFP in dendritic cells and macrophages labeled with CD11c or F4/80 antibodies, respectively, was confirmed by confocal microscopy.

Expression of membrane-bound HA was examined by staining cells with HA-specific monoclonal antibodies, followed by flow cytometry on a FACS Aria II flow cytometer.

**Confocal microscopy**

The cell cultures in CM were incubated on culture slides (SPL Life Sciences Ltd., S. Korea) suitable for the following microscopy. After incubation, the cells were fixed for 20 min in PBS containing 3.7% paraformaldehyde (Sigma), washed in PBS with 0.5% FBS, and then stained with antibodies for 1 h. After an extra wash with PBS, the cells were stained with DAPI (1 μg/ml) in PBS for confocal microscopy on a Axio Observer. Z1 microscope (Carl Zeiss, Germany) with a QuantEM 512SC camera (Photometrics, UK).

**Statistical analysis**

Data are presented as means + SD. Statistical analysis of the data was performed using Student’s t-test

## RESULTS

**Immunomax enhances expression of the protein encoded by rAd**

**Fig. 1:**
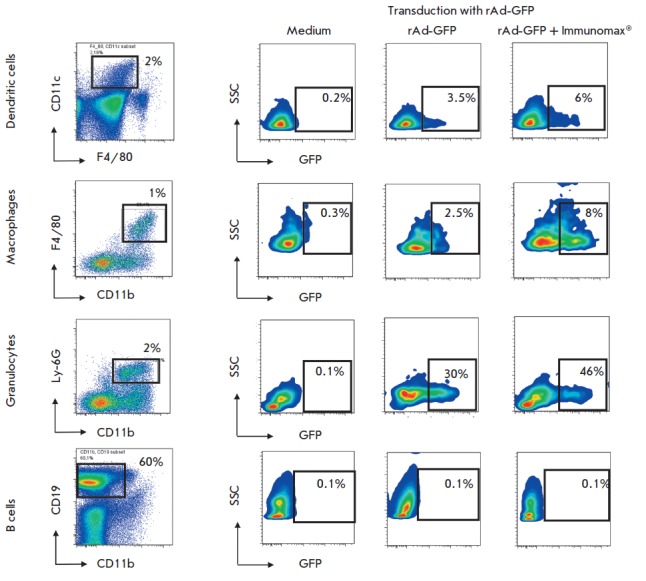
Influence of the TLR4-agonist (Immunomax) on the transduction and expression of rAd-GFP in different cell types of mouse spleen cells. Mouse splenocytes were transduced with rAd-GFP (5x105 PFU/ml) and incubated for 3 days in the presence of Immunomax (10 μg/ml) or its absence. Then cells were labeled with fluorochrome-conjugated antibodies and analyzed using a FACS Aria II flow cytometer. The left vertical –gating of CD11c^+^ dendritic cells, F4/80^+^ macrophages, CD11b^+^ Ly6G^+^ granulocytes and CD19^+^ B-cells with the indication of the cell type content (percent) in the total population of splenocytes. Respective horizontal lines represent the contents of GFP-positive dendritic cells, macrophages, granulocytes, and B cells after transduction with rAd-GFP and further cultivation with Immunomax or without. The negative control (medium) represents spleen cell cultures without transduction

**Fig. 2:**
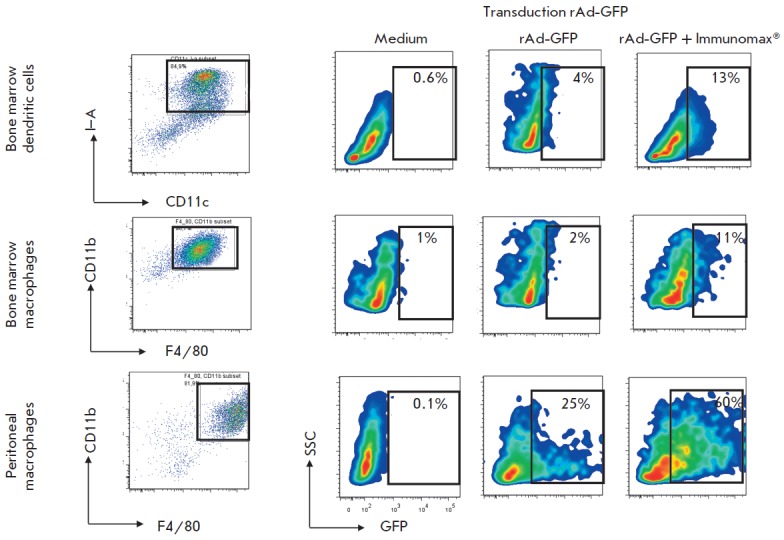
Influence of the TLR4-agonist (Immunomax) on the transduction and expression of rAd-GFP in dendritic cells obtained by the *in vitro *differentiation of mouse bone marrow cells, and also in mouse peritoneal macrophages. The cells were transduced with rAd-GFP (5x10^5^ PFU/ml) and incubated for 4 days in the presence of Immunomax (10 μg/ml) or its absence. Then, the cells were labeled with fluorochrome-conjugated antibodies and analyzed using a FACS Aria II flow cytometer. The left vertical –gating of CD11c^+^I-A^+^ dendritic cells and CD11b^+^F4/80^+^ bone marrow macrophages, and CD11b^+^F4/80^+^ peritoneal macrophages with the indication of these cell types percent in the total population. Respective horizontal lines represent the contents of GFP-positive dendritic cells and macrophages after transduction with rAd-GFP and further cultivation with Immunomax or without. The negative control (medium) represents cell cultures without transduction

**Fig. 3:**
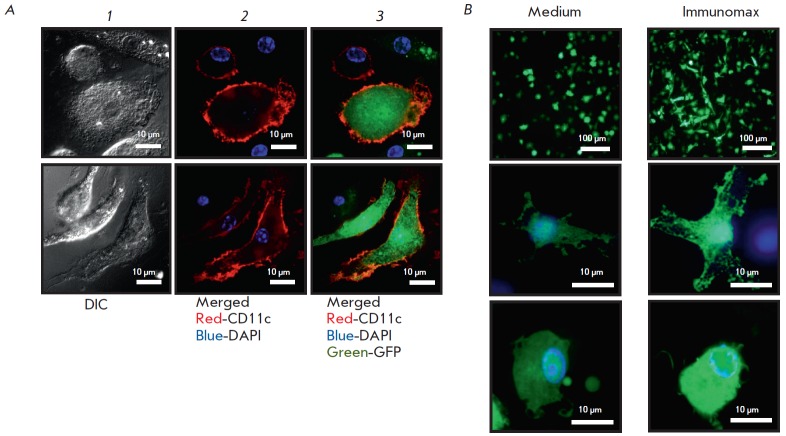
Confocal microscopy of dendritic cells transduced with rAd-GFP in the presence of Immunomax or its absence.** A **- cultures of bone-marrow dendritic cells incubated in a complete medium containing rAd (30 PFU per a cell). In 24 hrs, the cells were fixed and stained with the CD11c-PE antibody. Microscopy was performed in PBS with DAPI (1 μg/ml) using a Axio Observer. Z1 (Zeiss, Germany) with QuantEM 512SC camera (Photometrics, UK), by the use of 405 nm, 488 nm, and 561 nm lasers. From left to right, cell images are shown: (1) DIC –differential interference contrast; (2) merging of anti-CD11c-PE (red) and DAPI (blue) channels; (3) merging of GFP (green), DAPI, and anti-CD11c-PE channels.** B **–the culture of bone-marrow dendritic cells 24 hrs after incubation in the presence of rAd-GFP with Immunomax (10 μg/ml) or without. Photographs show the merged images in GFP (green) and DAPI (blue) channels at 20x and 200x magnifications

Replication-defective rAd readily transduce epithelial cells and transgene is efficiently expressed in this type of cells. For rAd-immunization purposes, it is critically important to achieve the target antigen expression within antigen-presenting cells, particularly dendritic cells and macrophages. In this study, we showed a substantial expression of rAd in murine primary dendritic cells and macrophages. Upon inoculation of rAd-GFP into cultures of spleen and bone marrow cells, as well as peritoneal macrophages, we observed the transgene expression in splenic dendritic cells, macrophages and granulocytes, and in bone marrow-derived macrophages and dendritic cells obtained in the presence of GM-CSF, and also in peritoneal macrophages (*Fig. 1 and 2*). No rAd expression was observed in lymphoid cells, in particular B-cells, CD4 and CD8 T-cells, and NK cells. The cell type expressing GFP was confirmed via staining with monoclonal antibodies to CD11c for dendritic cells, and F4/80 for macrophages, and Ly-6G for granulocytes. Figure 3A shows microphotographs of dendritic cells expressing CD11c molecules on their outer membrane and GFP in the cytosol.

**Fig. 4:**
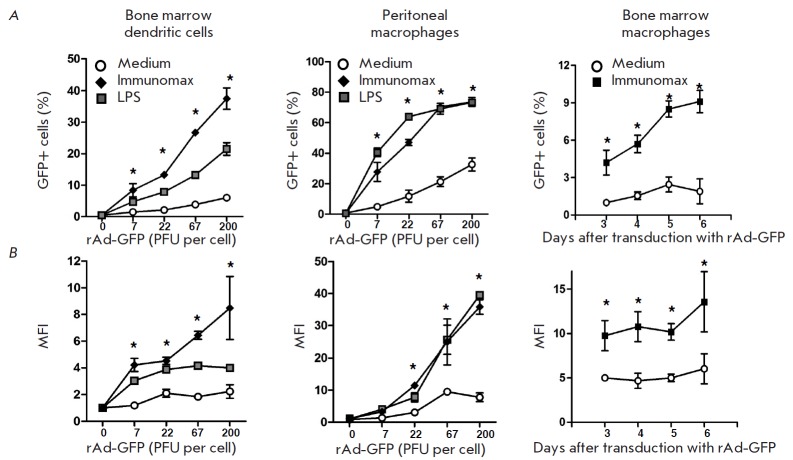
Influence of the TLR4 agonist (Immunomax) on GFP-expression in dendritic cells and macrophages depending on the rAd-GFP dose or the time elapsed after the transduction of cells. Bone marrow dendritic cells and peritoneal macrophages were transduced with rAd-GFP and then incubated for 24 hrs in the presence of Immunomax (10 μg/ml), or LPS (3 μg/ml), or without activators (medium). Bone marrow macrophages were transduced with rAd-GFP in the presence of Immunomax (10 μg/ml) or without it, and then they were incubated for 6 days. Cell samples were taken on days 3, 4, 5, and 6. After staining with fluorochrome-conjugated antibodies, the cells were analyzed using flow cytometry on a FACS Aria II. *x*-axis –rAd-GFP dose used for the transduction of cells, or days after transduction (the right side vertical). *y*-axis –(**A**) percent of GFP-positive cells; (**B**) mean fluorescence intensity (MFI) normalized on the value of the control cultures without the activator. Mean values and standard deviations are shown based on data from three experiments. Significance for P < 0.05 is shown using asterisk *

Activation of dendritic cells with Immunomax, simultaneously with transduction by rAd-GFP, led to enhanced expression of the GFP protein, which could be observed by an increase in both the proportion of GFPpositive dendritic cells (*Fig. 1, 2, 4A*) and the intensity of GFP production (*Fig. 3B, 4B*). A similar elevation in rAd-GFP expression was also observed in macrophages. Enhanced expression was observed regardless of the tissue origin of dendritic cells and macrophages. It was pronounced in dendritic cells from the spleen and bone marrow as well as macrophages from the spleen, bone marrow, or the peritoneal cavity (*Fig. 1–4*).

**No influence of Immunomax on rAd tropism for its target cell types**

When studying rAd expression in mouse spleen or bone marrow cell cultures, we noticed that Immunomax increased transgene expression in only those cell types that exhibited the vector expression in the absence of Immunomax. Immunomax did not retarget the adenovirus vector to different cell types. In particular, rAd-GFP was expressed in dendritic cells and macrophages but not in CD4 or CD8 T-cells, B-cells and NKcells. Immunomax increased rAd-GFP expression only in dendritic cells, macrophages, and granulocytes. In other cell types, the vector still showed no expression. As an example, Fig.1 illustrates B-cell studies (*Fig. 1*) in which rAd-GFP exhibited no expression both before and after activation with Immunomax.

**No influence of Immunomax on rAd replication in HEK-293 cells**

Enhanced expression of rAd-encoded transgene under the influence of Immunomax raises natural concern whether Immunomax can enhance also the replication of adenoviral particles. Since replication-defective adenovirus vectors cannot replicate in common cells, their propagation is usually achieved in the specially constructed HEK-293 cell line bearing adenoviral E1- genes, which are deleted from adenovirus vectors.

We examined if Immunomax affects the replication capacity of rAd in a HEK-293-TLR4/MD2 cell culture. In our previous studies, we had demonstrated that Immunomax acts through the TLR4 pathway, triggering the synthesis of reporter protein in HEK-293-TLR4/ MD2 cells. This effect of Immunomax is suppressed by CLI-095, a selective inhibitor of the TLR4-signal pathway. As with HEK-293 cells, HEK-293-TLR4/ MD2 cells exhibit active rAd replication, resulting in cytopathic effects on days 2–4. The rAd-GFP vector was titrated on a HEK-293-TLR4/MD2 cell culture in the presence or absence of Immunomax. The rAd-GFP vector was added in three wells of a 96-well plate at 40–50% confluence of HEK-293-TLR4/MD2 cells. Virus titration was performed as 24 steps of 5-fold successive dilutions. Cell cultures positive for rAd replication displayed intracellular GFP accumulation and cell death within a few days. The rAd vector replicated starting from the highest concentration to the 13th successive dilution. In the presence and absence of Immunomax (control cultures) the lowest rAd-GFP dilution at which GFP was expressed and cytopathic effects were observed was 513; i.e., Immunomax did not affect rAd replication in HEK-293-TLR4/MD2 cells carrying TLR4 receptors and triggering NF-κB activation in response to Immunomax.

**Immunomax enhances the expression of transgenes encoding cytoplasmic, secretory, or membrane-bound proteins.**

It was shown above that Immunomax enhances the expression of transgene incorporated in rAd, which encoded the cytoplasmic protein GFP. Here we demonstrate that the expression of secretory and membrane-bound proteins is also up-regulated in response to Immunomax. For these experiments, we used rAd-SEAP and rAd-HA, which encode the embryonic alkaline phosphatase and the influenza virus hemagglutinin, respectively. SEAP expression was estimated by its concentration in the culture medium, and HA expression was measured on the cell surface using flow cytometry with HA-specific monoclonal antibodies.

**Fig. 5:**
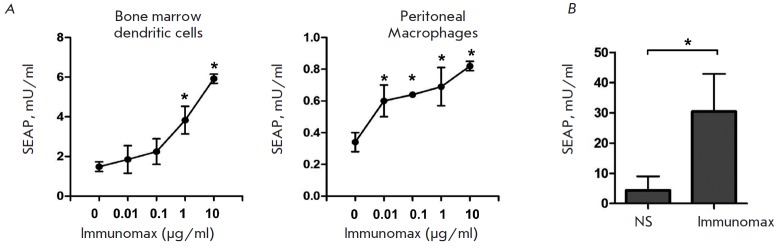
Influence of the TLR4 agonist (Immunomax) on the expression of the rAd-encoded secretory target protein (SEAP) in the *in vitro *cell cultures and in the mouse organism. (**A**). Mouse bone marrow dendritic cells and peritoneal macrophages were transduced with rAd-SEAP (5x105 PFU/ml) and incubated during 4 days in the presence of the shown concentrations of Immunomax. Production intensity of the target protein was estimated according to the concentration of SEAP (mU/ml) in culture supernatants. (**B**). The SEAP target protein concentration in mouse blood 3 days after injection of rAd-SEAP with Immunomax or without it. The experimental mice (n=4) were intraperitoneally injected with rAd-SEAP (108 PFU/mouse) with Immunomax (10μg) in a volume of 200 μl physiological saline. The control mice (n=4) were injected with a 200 μl volume of physiological saline, instead of Immunomax. Three days later, the concentration of SEAP was measured in the blood serum of all mice. Significance for P < 0.05 is shown using asterisk *

*Fig 5A *shows the expression of rAd-SEAP *i*n mouse dendritic cells and peritoneal macrophages. The findings indicate that Immunomax amplifies expression of the secretory protein SEAP encoded by rAd- SEAP. In studies of human monocytes transduced with rAd-HA, Immunomax also enhanced expression of the membrane-bound protein HA encoded by the vector. Of note, the increase in transgene expression was observed not only in mouse, but also in human cells.

**Immunomax enhances expression of rAd not only** *in vitro* **but also** *in vivo*

Our principal concern was to know whether the expression of rAd-encoded transgene could be enhanced* in vivo*. An increase in the target protein expression would be of great benefit to both immunization and gene therapy using rAd.

We investigated the influence of Immunomax on transgene expression in BALB/c mice that received rAd-SEAP intraperitoneally at a dose of 108 PFU in a 200 μl normal saline solution. The expression levels of SEAP were assessed by its blood concentration on day 3 post-injection. Experimental mice were injected with rAd-SEAP and Immunomax (10 μg/mouse). The control mice received rAd-SEAP with normal saline solution. The findings in *Fig. 5B *demonstrate that Immunomax caused a statistically significant increase in production of SEAP in mice injected with the rAd- SEAP vector.

**Enhanced rAd-expression in antigen-presenting cells is induced by agonists of TLR2, 4, 5, 7/8 and 9. The TLR3 agonist suppresses rAd expression**

**Fig. 6:**
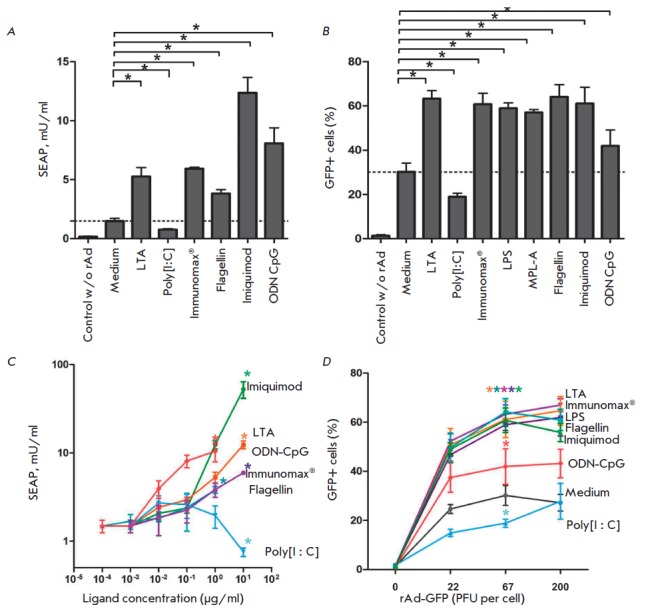
Influence of different Toll-receptor agonists on the transduction and expression of rAd-SEAP and rAd-GFP in mouse macrophages. Mouse peritoneal macrophages were transduced with (**A**, **C**) rAd-SEAP 5x107 PFU/ml, or (**B**) rAd-GFP 5x10^7^ PFU/ml, or (**D**) different concentrations of rAd-GFP, then the cells were incubated for 4 days in the presence of different TLR agonists. At the end of incubation, the concentration of SEAP in the culture supernatants (**A**,** C**), or percentage of GFP-positive cells (**B**, **D**) was determined. The following ligands were used in the experiments (**A**, **B**, **D**): LTA (1 μg/ml), poly[I:C] (10 μg/ml), LPS (10 μg/ml), MPL-A (5 μg/ml), flagellin (1 μg/ml), imiquimod (1 μg/ml), ODN-CpG 1826 (10 ng/ml), Immunomax (10 μg/ml). *x*-axis –the concentration of ligands in the experiments (**C**). Mean values and standard deviations are represented. Significance for P < 0.05 is shown using asterisk *

Immunomax acts as a TLR4 agonist which up-regulates rAd transgene expression in dendritic cells and macrophages. We were curious if another TLR4 agonist, in particular LPS, could exhibit the same activity. In addition, it was worthwhile to study agonists of other TLRs by their possible effects on rAd-encoded transgene expression. Data presented in *Figures 4 *and* 6 *confirm that, similarly to Immunomax, LPS amplifies the transgene expression. Interestingly, the monophosphoryl lipid A (MPL-A), a minimal immunostimulatory derivative of LPS, also enhanced rAd-GFP expression. In addition, agonists of TLR2, 5, 7/8, and 9, similarly to agonists of TLR4, up-regulated the expression of *SEAP* and *GFP *genes comprised in rAd-SEAP and rAd-GFP, respectively (*Fig. 6*). The transgene expression increase caused by agonists of TLR2, 4, 5, 7/8, and 9 ranged in different experiments from 2- to 11-fold (*P* < 0.05).

**Fig. 7:**
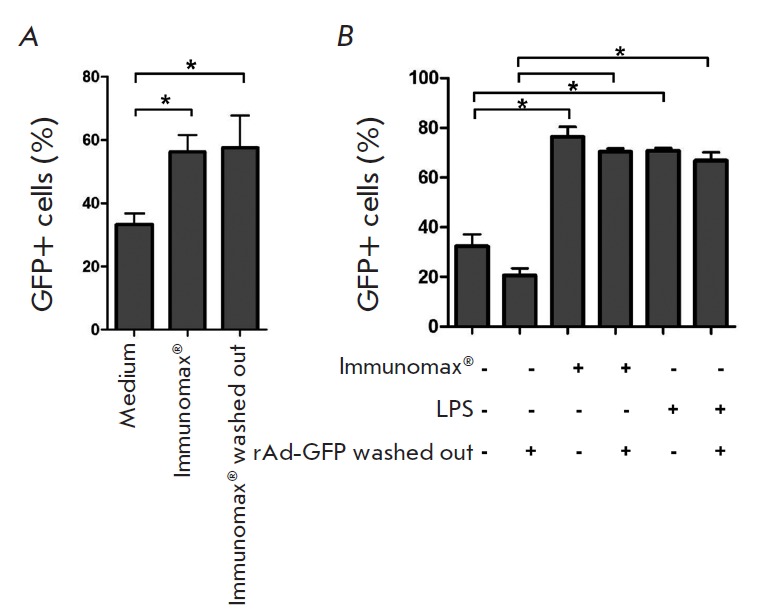
Enhancement of rAd-GFP expression by the sequential (separate) use of the rAd-GFP and TLR4- agonists. (**A**). Peritoneal macrophages (2x10^4^ per a well) were incubated during 4 hrs in a complete culture medium in the presence of Immunomax (10 μg/ml), then they were washed 3-times with PBS, transduced with rAd-GFP (70 PFU/cell), and transferred in the culture condition for further incubation under 37°C in 5% CO_2_. After 24 hrs, the culture medium was replaced with a Versene solution (PanEco), the cultures were kept for 1 hr at 4oC, then the cells were carefully washed and harvested in PBS (0.5% BSA) and analyzed for GFP-positive cells using a FACS Aria II flow cytometer. (**B**) Peritoneal macrophages (2x10^4^ per a well) were incubated during 2 hrs in a complete culture medium in the presence of rAd-GFP (70 PFU/cell). Then some of the wells were washed 3-times with PBS and re-filled with the complete culture medium with Immunomax (10 μg/ml) or LPS (3 μg/ml). Negative control cultures were washed and re-filled with the complete culture medium without activators. After 24 hrs, the cells were harvested using a Versene solution and analyzed for GFP-positive cells using a FACS Aria II flow cytometer

Importantly, increased rAd expression induced by agonist of TLR was not attributed to the complex formation between rAd and the agonists or facilitated up take of rAd. This was shown in experiments in which rAd was washed prior to adding TLR4 agonists (Immunomax, LPS) over a cell monolayer, or *vice versa*, the TLR4 agonist was washed away prior to transduction of cells with rAd. In both cases, the increase in rAd expression was consistent with that observed for simultaneous use of rAd and a TLR agonists (*Fig. 7A,B*).

It was unexpectedly found that in contrast to agonists TLR2, 4, 5, 7/8, and 9, the agonist of TLR3 not only did the up-regulation of rAd-expression, but it suppressed the production of protein encoded by rAd. Agonist of TLR3 inhibited expression of rAd-SEAP and rAd-GFP (*Fig.6*).

**NF-κB activation bypassing the TLR pathway also enhances transgene expression**

All TLRs, except for TLR3, mediate the intracellular signal through an adaptor protein, MyD88, which ultimately leads to activation of NF-κB. We suggested that transgene expression is up-regulated by TLR agonists due to the activation of the MyD88 → NF-κB signaling axis. This suggestion was verified using two approaches: inhibition of the signal tranduction from TLR to MyD88 and activation of NF-κB without engagement of TLR. Signal transduction from TLR4 to MyD88 was blocked using a selective inhibitor: CLI-095. Activation of NF-κB, bypassing TLR, was performed with TNF-α, which activates NF-κB via TNF receptors.

**Fig. 8:**
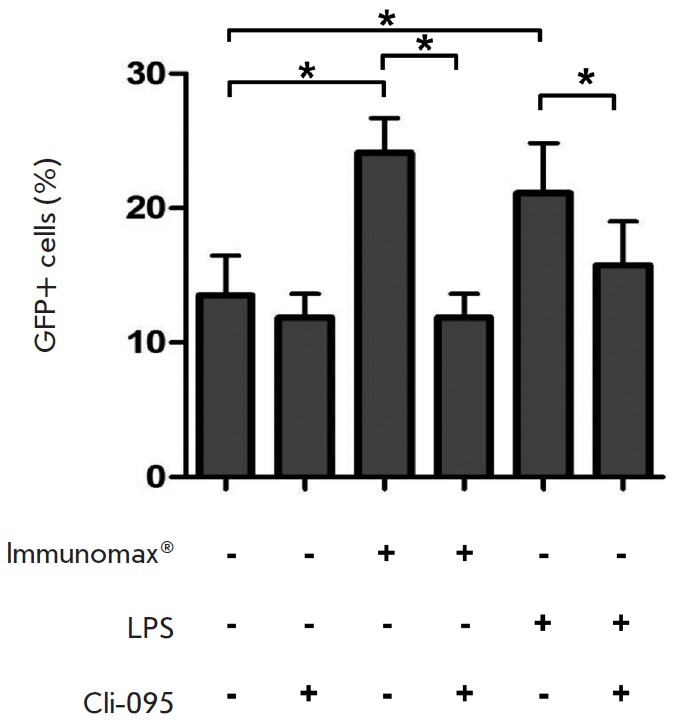
A selective inhibitor of TLR4-signal (CLI-095) abrogates the enhancement of rAd-GFP expression by the TLR4-agonists. Peritoneal macrophages were incubated for 1 hr in a complete culture medium containing CLI-095 (1 μg/ml) or without it, then rAd-GFP (70 PFU/ml) and Immunomax (10 μg/ml), or LPS (3 μg/ml) were added into the culture medium. Control cultures were transduced with rAd-GFP without activators. After 24 hrs, the cells were harvested using a Versene solution and analyzed for GFP-positive cells using a FACS Aria II flow cytometer

**Fig. 9:**
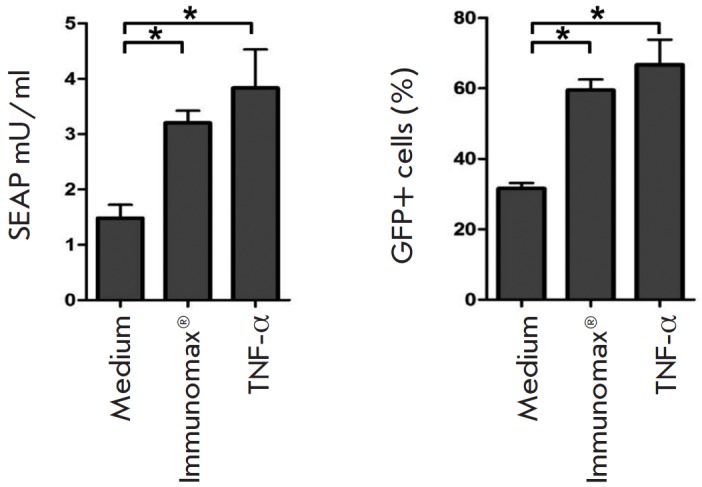
TNF-α enhances the expression of target proteins in macrophages transduced with rAd with inserts of the GFP- or SEAP-target genes. Mouse peritoneal macrophages were transduced with rAd-SEAP (5x10^7^ PFU/ cell) or rAd-GFP (5x10^7^ PFU/cell) then incubated during 4 days in the presence of Immunomax (10 μg/ml) or TNF-α (10 ng/ml). The control cultures were transduced with vectors in the absence of activators. At the end of incubation, the concentration of SEAP in the culture supernatants or percentage of GFP-positive cells was measured

The findings are given in *Figs. 8 *and *9. *As anticipated, CLI-095 abrogated the enhancement of rAd-GFP expression caused by Immunomax or LPS (*Fig. 8*). Activation of peritoneal macrophages with recombinant TNF-α (10 ng/ml) induced increase in the expres sion of *SEAP *and *GFP *(*Fig. 9*), similar to that caused by Immunomax. The latter indicates that activation of TLR-pathways is not necessary, while activation of NF-κB is sufficient for the enhanced expression of transgene encoded by rAd.

## DISCUSSION

Recombinant replication-defective adenovirus vectors transduce epithelial cells as well as dendritic cells and macrophages. The last two cell types are professional antigen-presenting cells, thus they are of interest in terms of rAd-based vaccines. Expression of rAd-encoded antigens in antigen-presenting cells ensures success of vaccines based on rAd. To elicit potent humoral and cell-mediated immune responses to the protein antigen at least two crucial requirements should be met. Firstly, the antigen-presenting cells should express target antigen peptide fragments bound to MHC class I and II molecules. Secondly, these cells also should express co-stimulatory CD80, CD86, and CD40 molecules on their surface, priming T-cells while encountering an antigen-presenting cell. The first requirement is met when the target antigen is produced by dendritic cells and macrophages transduced with rAd. To comply with the second requirement dendritic cells and macrophages must be activated through TLR receptors or other pathways.

In this work, we showed that the proteins encoded by rAd vectors are expressed in dendritic cells and macrophages (*Fig. 1–3*). Additional activation of antigen- presenting cells using the TLR4 agonist induces overexpression of the co-stimulatory molecules CD80, CD86, and CD40. In addition, as demonstrated in this study, the TLR4 agonist enhances the expression of the target protein (*Fig. 1–4). *An enhanced production of the protein antigen, together with expression of the co-stimulatory molecules CD80, CD86 and, CD40, could enhance efficacy of immune response when the combination of rAd and TLR4-agonist is used. We have previously reported that coadministration of rAd-HA encoding the influenza virus hemagglutinin and the pharmaceutical TLR4 agonist (Immunomax) allows one to increase efficacy of the vaccination against influenza viruses A and B [7].

Replication-defective adenovirus vectors could be used not only for vaccination, but also for gene therapy. In the latter case, administration of rAd with the transgene insert to a patient leads to the following production of the therapeutic protein during a period of 2-3 weeks. The possibility, shown in this study, for the increase of rAd-encoded protein production using combined administration of TLR4-agonist might be important for advancing effectiveness of rAd-based gene therapy. It is likely that the combination of rAd and TLR4-agonists enables one to obtain much higher concentrations of the therapeutic protein, as compared to rAd used alone, or substantially decrease the dose of rAd necessary to obtain the desired concentration of the protein.

TLR signaling has been the focus of much research for the last 15 years. These studies led to a good understanding of the intracellular signaling events triggered by ligands of TLR1/2, TLR2/6, TLR3, TLR4, TLR5, TLR7/8, and TLR9 in mouse and human cells [13, 14]. As was discovered, TLRs operate through two signaling pathways. One pathway is begun by the MyD88 key adaptor molecule and is ended by the activated NF-κB transcription factor. The other pathway is started by the TRIF key adaptor molecule and is ended by IRF transcription factors, in particular IRF3.

The signal pathway MyD88 → NF-κB comes into play when agonists act via TLR1/2, TLR2/6, TLR5, TLR7, TLR8 and TLR9 receptors. The signal pathway TRIF → IRF is employed during TLR3 activation. A unique feature of TLR4 is the use of both pathways. Immediately after ligation of the agonist, TLR4 signals from the outer cell membrane to the MyD88 → NF-κB pathway. Shortly, following endocytosis of the ligand-receptor complexes TLR4 initiates the second signal pathway TRIF → IRF.

In this study, we found that agonists of different TLRs enhance production of proteins encoded by rAd. Enhanced expression of transgene was observed following the use of the TLR2, TLR4, TLR5, TLR7/8 and TLR9 agonists (*Fig. 6A,B*). The TLR3 agonist acted opposite to the other agonists tested. Exposure of dendritic cells and macrophages to Poly[I : C] during their transduction with rAd-GFP or rAd-SEAP lead to a significant suppression of the GFP and SEAP production, respectively (*Fig. 6A,B*). Comparing various intracellular signaling pathways mediated by different TLRs, we suggested that the activation of NF-κB is responsible for up-regulation while the activation of IRF is involved in the down-regulation of the rAd-encoded protein synthesis.

**Fig. 10:**
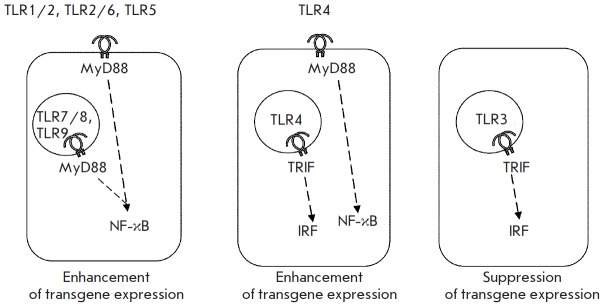
A hypothetical mechanisms of enhancement and inhibition of Ad-vector encoded target protein expression by different TLR-agonists

A special case is the TLR4 agonist. Since TLR4 agonists enhance production of rAd-encoded proteins, we suggest that the MyD88 → NF-κB signaling axis dominates over the TRIF → IRF one (*Fig. 10*).

The suggestion of a stimulatory role for the MyD88 →NF-κB signaling axis is partially confirmed by our findings. CLI-095, a specific inhibitor of signal transduction from TLR4 to the adaptor molecule MyD88, abrogated enhancement induced by TLR4-agonists in production of rAd-encoded protein (*Fig. 8*). In turn, NF-κB activation bypassing TLR caused amplified expression of transgenes encoded by rAd. Activation of cells with TN F-α simultaneously with their transduction with rAd-SEAP or rAd-GFP enhanced the production of SEAP and GFP, respectively (*Fig. 9*). TNF-α signaling is known to operate through the TNFR1 and TNFR2 receptors. The intracellular signaling pathway is ended by NF-κB activation. Hence, NF-κB activation bypassing TLR receptors also enhances transgene expression, which does not confirm but bolsters our suggestion that up-regulation of the transgene-encoded protein production is dependent on NF-κB.

The rAd-GFP, rAd-SEAP, and rAd-HA constructs used in this study contained genes of corresponding proteins under the control of NF-kB-responsive CMV promoter having four recognition sites for NF-κB [15]. It is logical to hypothesize that additional activation of the NF-κB by TLR2, TLR4, TLR5, TLR7, TLR8, and TLR9 agonists could promote transcription of genes under the control of CMV-promoter.

In principle, TLR-mediated signaling can enhance production of a protein by affecting transcription, translation, and other essential cellular processes. The precise mechanisms by which the transgene expression is up-regulated upon activation of TLR2, TLR4, TLR5, TLR7, TLR8, TLR9 or reduced by the activation of TLR3 remain to be elucidated.

Replication-defective adenovirus vectors are used not only for immunization, but also in gene therapy. The effects of TLR agonists on rAd-transgene expression reported herein hold promise for a new approach to developing controlled transgene expression techniques* in vivo*. Ideally, advances in this field would allow to develop methods for controlled up-regulation or down-regulation of transgene expression *in patient*, depending on the purpose.

## CONCLUSIONS

In our study, we examined the effects of Toll-like receptor agonists on the efficacy of transduction with and expression of rAd in the antigen-presenting cells of humans and animals. It is demonstrated that the agonist of TLR2, 4, 5, 7, 8, and 9 enhance production of the protein encoded by rAd. The enhancement occurs in dendritic cells and macrophages producing cytoplasmic (GFP), membrane-bound (HA), or secretory (SEAP) proteins. Experiments in mice showed that target protein expression can be also enhanced in the animal organism with the use of a pharmaceutical TLR4 agonist. In contrast to other TLR agonists, the TLR3 agonist suppresses production of the protein (GFP or SEAP) in cells transduced with rAd having a corresponding gene insert.

The molecular mechanisms of the up- and downregulation of rAd expression in antigen-presenting cells activated with various TLR agonists remain to be determined. In this paper, we reported on results that support the suggestion that the enhancement in rAd-transgene expression is due to the activation of the transcription factor NF-*k*B and that the suppression is attributed to the activation of IRF transcription factors.

## Abbreviations

rAd: replication-defective recombinant adenovirus vector
TLR: Toll-like receptor
PFU: plaque-forming unit
CM: complete culture medium
BSA: bovine serum albumin
PBS: phosphate buffered saline
LTA: lipoteichoic acid
Poly [I:C]: polyinosinic : polycytidylic acid
LPS: lipopolysaccharide
MPL-A: monophosphoryl derivative of lipid A
TNF-α: tumor necrosis factor alpha
GM-CSF: granulocyte macrophage colony stimulating factor
CLI-095: also known as TAK-242, a specific inhibitor of TLR4-signaling
SEAP: secreted embryonic alkaline phosphatase
GFP: green fluorescent protein
DAPI: 4′,6-diamidino-2-phenylindole dihydrochloride
HA: H1N1 influenza virus hemagglutinin
CMV: Cytomegalovirus
IL: interleukin
